# Use of telehealth to improve healthcare access and outcomes in surgical oncology

**DOI:** 10.1002/jso.27844

**Published:** 2024-08-27

**Authors:** Elliot A. Asare, Lauren Cowan, Tracy Onega

**Affiliations:** ^1^ Department of Surgery University of Utah Salt Lake City Utah USA; ^2^ Huntsman Cancer Institute University of Utah Salt Lake City Utah USA; ^3^ Department of Population Health Sciences University of Utah Salt Lake City Utah USA

## Abstract

The dimensions of healthcare access includes availability, accessibility, accommodation, affordability, and accessibility. Many patients face significant barriers to accessing oncologic care and subsequently, health outcomes are suboptimal. Telehealth offers an opportunity to mitigate many of these barriers to improve health access and outcomes. This review discusses how telehealth can be leveraged to improve healthcare access in surgical oncology while also highlighting important challenges to realizing the full potential of this mode of healthcare delivery.

## 1 BACKGROUND

Improving access to healthcare is critical to better health outcomes for individuals and populations. Healthcare access is multidimensional with many barriers to overcome to ensure that individuals and populations can harness the great advances made in preventative, curative intent, and palliative oncology to their advantage. The promise of telehealth as a tool to improve healthcare access is exciting, but it is critical for us to define “access” so that efforts at mitigating barriers can be targeted and measures of success and failure can also be established.

The definition of healthcare access as the “degree of alignment between characteristics of healthcare systems, and providers, and the characteristics, needs and expectations of patients and populations” is very appropriate for the purposes of this review.^1^ In their seminal work on “The Concept of Access,” Penchansky and Thomas outlined the following dimensions of healthcare access: availability, accessibility, accommodation, affordability, and acceptability.^1^ Availability and accessibility comprise the spatial domain of health access while accommodation, affordability, and acceptability are aspatial. Healthcare access is a foundational pillar in the discussion of health disparities defined by the United States Department of Health and Human Services as “differences in health outcomes that are closely linked with social, economic, and environmental disadvantage and are often driven by the social conditions in which individuals live, learn, work and play.”^2^ Despite successes at reducing cancer‐related deaths,^3^ there remain persistent and significant disparities in cancer outcomes. Most recent report showed that Black patients with prostate, gastric, and uterine corpus cancer had twofold higher mortality rates compared to White patients.^3^ Also, Native Americans with liver, gastric, and kidney cancer had twofold increased mortality compared to White patients.^3^ Data from the Centers for Disease Control and Prevention (CDC) shows that patients from nonmetropolitan rural counties have higher death rates for all cancer sites combined compared to nonmetropolitan urban and metropolitan counties.^4^

With this background information, we can examine how telehealth can be utilized to improve health access, mitigate health disparities, and ultimately improve health outcomes for all surgical oncology patients.

### 1.1 Telehealth and its scope

Telehealth is “the use of electronic information and telecommunication technologies to support long‐distance clinical health care, patient and professional health‐related education, health administration, and public health.”^5^ It is important to distinguish between telehealth and telemedicine as the latter focuses on the use of technologies to deliver medical care to patients who are in separate geographic locations from their clinicians. For example, during an episode of telemedicine care, a surgeon may review labs and discuss their implications with a patient. This telemedicine encounter together with other activities that the surgeon may participate in, such as virtual multidisciplinary treatment planning conferences and providing outreach education to other clinicians at a remote location, constitute telehealth.

### 1.2 Telehealth technologies and applications in surgical oncology

There are a plethora of tools available that can be used to meet telehealth needs. These technologies may be used to deliver telehealth in a synchronous or asynchronous manner.^5^, ^6^ Synchronous telehealth is real‐time engagement between clinicians and patients, such as patient follow‐up using a secure video portal. In asynchronous engagement, interactions between clinicians and patients are separated by time in addition to distance, such as reviewing photos of surgical wounds sent via secure email portal and following up later with patients to address their concerns.

The following categories of technologies can be leveraged to improve access to care: (i) internet, (ii) video, (iii) mobile health (mHealth), (iv) store‐and‐forward, (v) remote patient monitoring, and (vi) land and wireless communication.^5^, ^6^

### 1.3 Healthcare access barriers in surgical oncology and telehealth solutions

#### 1.3.1 Geographic availability and accessibility (spatial access)

One in six Americans live in a rural area, and 62% of the 3221 counties in the United States are rural. The distribution of the oncology and surgical workforce is uneven and overwhelmingly favors metropolitan centers of residence and work. Optimal delivery of cancer care requires multidisciplinary expertise which remains sparse in rural areas. Workforce data shows 10%, 11.6%, and 13% of general surgeons, medical oncologists, and radiation oncologists, respectively, practice in rural America, even though 17%–20% of the U.S. population resides in rural areas.^7^, ^8^, ^9^, ^10^ Specifically for complex general surgical oncology board‐certified surgeons, it is unknown what percentage practice in rural areas, but evidence shows a 29.1% reduction in the supply of general surgeons who practice in rural areas from 2001 to 2019.^7^ The availability of other cancer subspecialists such as head & neck (H&N) surgeons, urologic oncologists, orthopedic oncologists, thoracic surgeons, pathologists, and interventional radiologists would be expected to be even lower to non‐existent in some rural areas.^11^, ^12^ In fact, one study of the distribution of fellowship‐trained H&N surgeons from 2011 to 2020 found that Alaska, Idaho, Wyoming, North Dakota, and Mississippi did not have any.^13^ While availability deals with distribution of oncology workforce and resources relative to the population served, accessibility is the travel time or distance needed to reach clinicians and services.^1^, ^14^ It has been shown that the travel times to National Cancer Institute (NCI)‐designated cancer centers, academic medical centers, and oncologists is considerably longer for Native Americans and nonurban dwellers.^15^

#### 1.3.2 Use of telehealth to mitigate spatial barriers to healthcare access

While multipronged strategies are needed to increase the presence of oncology clinicians in rural areas, telehealth can be used to mitigate some of the challenges posed by lack of clinician availability on site. Video technology is now being used widely to facilitate multidisciplinary cancer conference discussions. This asynchronous use of telehealth allows experts from different specialties around the world to discuss the best treatment options for patients who would otherwise have had to travel many miles for in‐person consultations. The benefits of telehealth in multidisciplinary treatment planning conferences, such as ease of attendance for physicians, increased participation, and ability of remote sites with the right technology to participate and thus minimize delays in care, have been shown in several studies.^16^, ^17^, ^18^ Specifically for surgical oncology, secure video technology can be used for synchronous preoperative consultation, postoperative visits, and surveillance. A randomized control trial (RCT) comparing video visits with in‐person office visits for surveillance in patients who underwent prostatectomy for prostate cancer showed no differences in time devoted to patient care and no increased risk of acute presentation with urologic issues within 3 months of visit.^19^ The RCT by Viers et. also showed improved accessibility in the cohort randomized to video visits vs in‐person visits as evidenced by 0 versus 95 miles and 0 versus 95 min distance and time traveled respectively.^19^ Asynchronous use of telehealth through provision of high‐quality, well‐curated websites with information on perioperative educational materials can be very helpful to patients and save time, travel and money.

## 2 USE OF TELEHEALTH TO MITIGATE ASPATIAL BARRIERS TO HEALTHCARE ACCESS

The aspatial domains of healthcare access are comprised of accommodation, affordability, and acceptability. Factors such as hours of operation, ease of getting an appointment, ability to reach out to clinical team with concerns when appropriate all fall within “accommodation” which is defined as “the relationship between the manner in which the supply resources are organized to accept clients and the clients' ability to accommodate to these factors and the clients’ perception of their appropriateness.”^1^ For example, work demands may make it difficult for a farmer to take time off work to travel many miles for a follow‐up appointment with a surgical oncologist during peak harvest season. In this example, asynchronous use of telehealth services through “store‐and‐forward” technology may allow the surgeon to receive, review pertinent imaging and labs, and communicate his/her concerns to the patient via secure email using the patient portal. A video visit could be arranged at a convenient time for patients to allow better engagement. Remote patient monitoring with mobile apps, wearable devices, and computers may allow effective data tracking and potentially facilitate early detection of postoperative complications. In a study of over 12 000 patients who underwent ambulatory surgery at a single NCI‐designated cancer center in New York, Crachiolo et. al. characterized the pattern of postoperative recovery using a platform with automated clinical alerts for remote symptom monitoring.^20^, ^21^ The ability to remotely monitor patients spares them the burden of travel when they are convalescing and allows effective triage by a clinical care team. In a systematic review of 11 studies covering a broad spectrum of oncologic surgical specialties and complexity of procedures, Xiao et. al did not find increased rates of emergency readmissions or required in‐person visits for patients who had virtual postoperative visits.^22^ Additionally, there were no differences in 30‐day, 90‐day morbidity, and 90‐day mortality between patients seen via telemedicine and those seen in‐person.^22^ Results from seminal clinical trials form the backbone of guidelines and treatment recommendations, but the percentage of cancer patients who enroll in clinical trials is estimated to be about 5%.^23^, ^24^ Added to this overall low enrollment rate is that only 8%, 11%, and 6% of clinical trial participants are African American/Black, Latino/Hispanic, or Asian, respectively.^25^ In a survey conducted by the Association of Community Cancer Centers Community Oncology Research Institute, only 25% of rural oncology practices offered phase 1 trials, compared with 67% of practices located in urban areas.^26^ Barriers to clinical trials participation are multifactorial, but telemedicine can be leveraged to mitigate some of the spatial barriers faced by patients from racial minority groups, low socioeconomic backgrounds, and rural locations. In addition to stringent inclusion/exclusion criteria, most trials are centralized and require patients to present at designated locations for routine labs, physical exams, and other assessments. This increases the burden of travel with accompanying issues such as increased patient anxiety, inconvenience, missed work, and additional financial burden even if insurance covers the cost of trial participation. In a mixed‐methods study of 14 National Multi‐site Collaborative Cancer Clinical Trials Groups in Australia in which clinicians, nurses, allied health professionals, trial executives, and coordinators participated, a hybrid model of telehealth and in‐person visits was viewed most favorably.^27^ Participants reported that telehealth tools such as wearables, remote patient monitoring, video conferencing, and telephone calls could enhance the reach of clinical trials, improve efficiency, and advance equity.^27^

For access to quality health care, affordability cannot be overlooked. Nearly 10% of adults in the United States do not have health insurance.^28^ Even for those with health insurance, financial toxicity arising from the cost of cancer care is significant. Studies have reported that cancer patients have increased likelihood of experiencing home foreclosures, filing for bankruptcy, or undergoing repossessions compared to non‐cancer patients.^29^, ^30^ Minimizing travel or eliminating it, when possible, by using telehealth can lead to cost savings for patients and healthcare systems. Studies have shown that synchronous telemedicine or the use of remote symptom monitoring in a cohort of patients undergoing various oncologic surgeries was associated with reduction in potentially avoidable urgent care visits.^19^, ^21^ Also, for patients who must travel longer distances for care, the cost of temporary housing, fuel for cars, and food add to the burdens of healthcare access.^31^ Telehealth may decrease days missed from work for both patients and family members or friends that they must rely on as travel companions or drivers. In an economic evaluation analysis of the indirect cost savings (from lost productivity and travel to and from appointments) afforded by telehealth visits for patients treated at an NCI‐designated cancer center in Florida, there were substantial savings in roundtrip miles traveled, roundtrip hours traveled, and cost.^32^ In this analysis of 11,688 patients with cancer, the estimated mean total cost savings from telehealth visits ranged from $147.4 at $0.56/mile to $186.1 at $0.82/mile.^32^

The final aspatial domain of healthcare access is acceptability which is defined as “the relationship of clients' attitudes about personal and practice characteristics of providers to the actual characteristics of existing providers as well as to provider attitudes about acceptable personal characteristics of clients.”^1^ Patients may determine acceptability based on location of healthcare facility, perception of cleanliness of facility, perception of staff friendliness, quality of food in cafeteria, and so forth, while clinicians may view patient acceptability factors to include timeliness to appointment, demeanor, and interactions with staff, and so forth. In semi‐structured interviews of medical oncology patients treated at a single institution, positive factors that enhanced acceptability of telehealth visits included elimination of travel, reduction in anxiety, and longer visit with clinicians.^33^ In their RCT of prostatectomy patients, Viers et. al did not find any differences in perception of trust, visit confidentiality, efficiency, quality of patient education, or overall encounter satisfaction between telehealth visits and in‐person visits.^19^ Another survey study of cancer patients from Australia of 124 respondents found that 93% of patients were satisfied with their telehealth experience, and the majority felt that the quality of their telehealth experience was equivalent to in‐person visits.^31^ Similarly, survey results of patients with gynecologic malignancies in Australia showed high patient satisfaction with the following emerging as core themes: emotions, relationship building, communication, and acceptability.^34^

As healthcare systems, patients, and the broader world look for ways to mitigate the threat of climate change, telehealth can help reduce our carbon footprints by minimizing the frequency of travel for care. In a study of vascular surgery patients from the United Kingdom, telehealth reduced the carbon footprint of each patient by 41.2 (24.5–80.3) kgCO_2_e.^35^ In a cohort study of 123 890 cancer patients in the United States that compared in‐person‐preferred cancer care model with telemedicine‐preferred model, there was an 81% relative reduction in CO_2_ equivalent emissions during the COVID‐19 pandemic.^36^ These positive effects of telehealth on the environment is ultimately an enhancement to healthcare access.

Table 1 shows examples of how telehealth can be used to mitigate healthcare access barriers in surgical oncology.

**Table 1:** Dimensions of healthcare access, associated barriers and examples of telehealth uses to mitigate.

	Examples of barriers in domain	Examples of telehealth mitigation of barriers
Accessibility	Proximity of services ‐ distance and travel time	‐Secure video technology for synchronous consultation; Asynchronous provision of curated patient education/decision‐making materials
Availability	Distribution of oncology workforce: Supply of (sub)specialists	
Accommodation	Hours of operation; Ease of getting an appointment; Ability to reach out to clinical team with concerns	‐Asynchronous use of “store‐and‐forward” technology; ‐Video and teleconference visits; Remote patient monitoring
Affordability	Lack of insurance: Financial toxicity: Indirect costs of lost wages, travel costs, remote housing	
Acceptability	Timeliness to appointment; Staff interactions/demeanor; Health care facility location, cleanliness, and so forth.	

### 2.1 Barriers to telehealth implementation and utilization

Although telehealth offers many genuine advantages that may improve health access and ultimately outcomes, there are formidable barriers to implementation and optimal utilization as shown in Table 2. The barriers to optimal telehealth access are disproportionately borne by populations that have traditionally experienced health disparities: racial and ethnic minorities, rural patients, elderly patients, patients with disabilities, and those with low socioeconomic status (SES). Several studies have reported lower rates of telehealth utilization among older patients, rural patients, racial/ethnic minorities, and those of low SES.^37^, ^38^, ^39^, ^40^

**Table 2:** Barriers to telehealth implementation and utilization and strategies to improve telehealth access.

**Barriers**
Digital access
Digital literacy
Telehealth access points and network security
Sensory and cognitive barriers
Surgeon/provider barriers
**Strategies**
Governmental investments in broadband connectivity
Telehealth access points in public locations
Investment in cybersecurity and interoperability
Increase state licensure reciprocity
Increase coverage and reimbursement for services

#### 2.1.1 Digital access

Access to broadband internet and electronic devices such as desktop computers, laptops, or smartphones are critical to telehealth. While there has been a steady increase in the number of individuals and households that have access to these digital tools, the data shows that some demographics lag. Data from Pew research organization showed that home broadband internet access in 2023 was 68% and 83% for Black and White households, respectively, in 2023.^41^ Homes with income < $30 000 reported 57% broadband access compared to 92% for households with income of $100 000. A similar pattern of lower rates of home broadband access was noted in rural communities, homes with less than high school graduation, and ages ≥65 years old.^41^ In a study to examine access to high‐speed internet via desktop computer, laptop computer, smartphone with a data plan, or any other digital access that involved 1 210 714 respondents from the American Community Survey, 14.02% of households reported that they did not have any digital access.^42^ Another study using the American Community Survey found lower rates of broadband internet access in geographically isolated areas with higher population of Black, American Indian/Alaska Native, and Hispanic households.^43^

#### 2.1.2 Digital literacy

Having the knowledge or support to set up and troubleshoot the device (laptop, computer, smartphone) for accessing telehealth is crucial to allow the telehealth encounter to occur, facilitate quality encounters, and minimize frustrations. For synchronous telemedicine encounters, patients are expected to be able to login to a secure portal and ensure that their camera and audio on a digital device are working appropriately. Sometimes a patient may not be able to locate the portal to login or a glitch may occur where audio may work but camera does not or vice versa. The level of digital literacy has been shown to correlate with telehealth adoption with the elderly, low SES, and less educated being on the low end of the telehealth adoption spectrum.^44^ For asynchronous telemedicine encounters, patients who do not speak or understand English are disadvantaged as the system is not set up to include interpreter services in such scenarios. Immigrant populations who would otherwise benefit greatly from telehealth for the reasons previously outlined may not be able to do so due to language barrier or the difficulty of integrating interpretive services into asynchronous telemedicine encounters.

#### 2.1.3 Telehealth access points and network security

Whether via video or audio, synchronous telehealth requires privacy to allow physical exams and discussion of sensitive information without compromise as would have been done in person. Lack of privacy due to living in crowded households or being homeless will present significant challenges to optimal telehealth access and utilization. In addition, data breaches from hacking can present serious threats to telehealth due to identity theft, blackmail, and extortion. There has been an increase in cases where cyber criminals extort healthcare systems by demanding ransom payments. In such situations, affected networks are shut down, preventing patients from utilizing telehealth to meet their healthcare needs. Unfortunately, as healthcare becomes more digitized, cybersecurity threats will continue to increase with consequences for telehealth.^45^

#### 2.1.4 Sensory and cognitive barriers

Some individuals with visual, hearing, speaking, or cognitive impairment may encounter physical barriers to digital devices such as desktop computers, laptops, and smartphones needed to access telehealth services. Elderly patients are more likely to have undiagnosed impairments leading to difficulties with telehealth visits. In a study to assess telerehabilitation readiness among older adults in the United States using survey data from the National Health and Aging Trends, sensory and cognitive impairment were among the main drivers of telehealth unreadiness.^46^ Difficulties in hearing, seeing, or understanding during a telehealth encounter can frustrate both patients and clinicians, diminishing satisfaction for both parties.

#### 2.1.5 Surgeon/clinician barriers

There are intrinsic and extrinsic factors that may influence a surgeon's participation in telehealth. Intrinsic factors include digital literacy and workflow habits. Although well‐educated and technically skilled in the operations they perform, it cannot be assumed that all surgeons are digitally literate or enjoy using digital tools. Time constraints may hinder surgeons' ability to participate in telehealth training services which are often provided through the Information Technology (IT) department. Without adequate training to optimize and maximize digital tools for telehealth encounters, frustrations can lead to a vicious cycle of avoidance of telehealth use. In addition, without adequate support, some surgeons may find telehealth disruptive to their well‐established workflow and thus avoid using it if possible.

Extrinsic barriers to surgeons' adoption of telehealth include medical licensing, reimbursement, and inability to perform certain physical exams. Most physicians are licensed in the state in which their practice is physically located. In the United States, medical licenses are issued state by state, and, therefore, physicians seeking to practice in multiple states via telehealth must obtain medical licenses for each state. The administrative burden and financial cost of licensing process can deter physicians from offering telehealth services to patients. It is also critical that physicians are adequately reimbursed for services rendered via telehealth. During the COVID‐19 pandemic, Centers for Medicare and Medicaid Services (CMS) allowed Medicare reimbursements for telemedicine via video at par with in‐person visits, but there is concern as to how long this payment parity will continue in the post‐pandemic era.^47^ Also, the limitation of reimbursement for telemedicine to video alone presents a significant barrier to broad access for situations where audio may be the only available modality. Finally, without the physical presence of a patient, physical exam is substituted by visual inspection which may be inadequate.

## 3 STRATEGIES TO IMPROVE TELEHEALTH ACCESS

To improve healthcare access through telehealth, concerted efforts involving individuals, institutions, and government are necessary.

Federal and state government investments in improving broadband internet and 5G network access is critical in this endeavor. In 2018, the Federal Communications Commission (FCC) launched the “Connect America Fund” to expand broadband internet access to over 700 000 homes and businesses in rural areas.^48^ This program has allocated $1.488 billion to be distributed over 10 years to expand broadband access in areas that otherwise would not have them. The $9 billion “5G Fund for Rural America” was also established in 2020 by the FCC to expand 5G wireless network service to rural areas.^49^ These endeavors will help patients living in rural communities to access telehealth services.

Homelessness and housing insecurity have significant negative effects on health outcomes.^50^ To mitigate some of the challenges to telehealth access in this population, establishing “telehealth access points” within well‐established business locations, such as grocery stores, gas stations, shopping malls, or banks may be reasonable. These safe locations would offer the privacy and digital tools needed to access telehealth services for those who may not have homes or lack the privacy in their dwellings.

Investments in cyber security by governments and institutions are crucial to battle the rising threat to digital health by cyber criminals. It is also important to educate patients and healthcare employees about practices to safeguard data on the world wide web. Additionally, pursuing innovative technologies that can withstand or evade cyber security threats will be helpful. It is hoped that telehealth experiences will be greatly enhanced in the future as tremendous advances are made in developing the metaverse and integrating it into digital health tools. Defined as “a persistent virtual environment that allows access to and interoperability of multiple individual virtual realities,”^51^ the metaverse would allow digital twinning where the surgeon could superimpose CT scans or MRIs on the digital twin of patient to educate them about the location of their tumor, discuss planned surgery, and perhaps simulate potential outcomes. The limitations of physical examination with current telehealth technology may be overcome with sensory teleportation.^52^

Great leaps have been made in artificial intelligence (AI) over the last decade with exciting innovations in generative AI across many disciplines. As AI increasingly becomes readily available through incorporation into routine digital tools such as laptops and cell phones, it is anticipated that it would be leveraged to improve healthcare and healthcare access. For example, AI may facilitate easy language translation in both synchronous and asynchronous telehealth encounters. Well‐trained and validated models may be used to enhance health literacy, help patients navigate telehealth services, and facilitate remote patient monitoring and data collection as part of telehealth.

While physicians are required to be licensed in each state, the Interstate Medical Licensure Compact has streamlined the process for physicians to obtain licensing in multiple states and currently includes 37 states, the District of Columbia, and the Territory of Guam.^53^, ^54^ Policies that promote medical license reciprocity among all states would help improve patients' access to telehealth.

Finally, the CMS authorized payment parity for telehealth services during the COVID‐19 pandemic.^47^ This authorization ensures that physicians are reimbursed at the same rate as in‐person visits, and this protection has been extended through end of December 31, 2024. Reimbursing physicians adequately for services rendered via telehealth will ensure that they continue to offer this option to patients when appropriate.

## 4 CONCLUSION

Telehealth has the potential to mitigate health care disparities and improve outcomes in surgical oncology through enhanced health access. A thoughtful, multifaceted approach is needed to enable us to harness the full potential of this mode of healthcare delivery.

## Synopsis

Many patients face significant barriers to accessing oncologic care and subsequently, health outcomes are suboptimal. Telehealth offers an opportunity to mitigate many of these barriers to improve health access and outcomes.

## Data Availability

Data sharing not applicable‐no new data generated. Data sharing is not applicable to this article as no new data were created or analyzed in this study.
